# Spatial spillover effect of environmental factors on the tuberculosis occurrence among the elderly: a surveillance analysis for nearly a dozen years in eastern China

**DOI:** 10.1186/s12889-024-17644-5

**Published:** 2024-01-17

**Authors:** Dan Luo, Luyu Wang, Mengdie Zhang, Leonardo Martinez, Songhua Chen, Yu Zhang, Wei Wang, Qian Wu, Yonghao Wu, Kui Liu, Bo Xie, Bin Chen

**Affiliations:** 1 Department of Public Health, Hangzhou Medical College, Hangzhou, Zhejiang China; 2 School of Urban Design, Wuhan University, Hubei Wuhan, China; 3 Zhejiang University School of Public Health, Hangzhou, Zhejiang China; 4 Department of Epidemiology, School of Public Health, Boston University, Boston, MA USA; 5 Department of Tuberculosis Control and Prevention, Zhejiang Provincial Center for Disease Control and Prevention, Hangzhou, Zhejiang China; 6 Department of Public Health, Zhejiang University School of Medicine, Hangzhou, Zhejiang China 310058; 7 National Centre for Tuberculosis Control and Prevention, Chinese Center for Disease Control and Prevention, Beijing, China

**Keywords:** Pulmonary tuberculosis, Spatial-temporal analysis, Environmental exposure, Spatial Durbin Model

## Abstract

**Background:**

In many areas of China, over 30% of tuberculosis cases occur among the elderly. We aimed to investigate the spatial distribution and environmental factors that predicted the occurence of tuberculosis in this group.

**Methods:**

Data were collected on notified pulmonary tuberculosis (PTB) cases aged ≥ 65 years in Zhejiang Province from 2010 to 2021. We performed spatial autocorrelation and spatial-temporal scan statistics to determine the clusters of epidemics. Spatial Durbin Model (SDM) analysis was used to identify significant environmental factors and their spatial spillover effects.

**Results:**

77,405 cases of PTB among the elderly were notified, showing a decreasing trend in the notification rate. Spatial-temporal analysis showed clustering of epidemics in the western area of Zhejiang Province. The results of the SDM indicated that a one-unit increase in PM_2.5_ led to a 0.396% increase in the local notification rate. The annual mean temperature and precipitation had direct effects and spatial spillover effects on the rate, while complexity of the shape of the greenspace (SHAPE_AM) and SO_2_ had negative spatial spillover effects.

**Conclusion:**

Targeted interventions among the elderly in Western Zhejiang may be more efficient than broad, province-wide interventions. Low annual mean temperature and high annual mean precipitation in local and neighboring areas tend to have higher PTB onset among the elderly.

## Background

Tuberculosis (TB) is one of the deadliest infectious diseases, causing substantial concern globally. The single agent of this disease, *Mycobacterium Tuberculosis* (MTB), is transmitted by the respiratory tract causing lesions in nearly all tissues and organs [1]. In 2022, an estimated 10.6 million people worldwide fell ill with TB, with an incidence of 133 cases per 100,000 people and 1.3 million deaths [1]. Although continuous efforts had been implemented in high burden countries, China, as one of 30 high burden countries, still accounts for 7.1% of global cases [1]. Among the affected population, the elderly group had contributed to a substantial part [2]. Along with aging, influencing factors such as the decreased immune function, cognitive deficiency, and insufficient social and family care might also lead to increased morbidity and PTB transmission risk in this special group [3, 4]. Thus, increasing attentions was directed on lowering the reactivation of latent pulmonary TB (PTB) infection and preventing new infections among the elderly.

Increasing evidence demonstrated that the dominated drivers of PTB development not only included personal factors such as gender, malnutrition, Acquired Immune Deficiency Syndrome (AIDS), diabetics, smoking and alcohol consumption, but were also associated with environmental factors and spatial locations [5–7]. For environmental factors, available research showed that atmospheric pollutants such as PM_2.5_ and SO_2_ were associated with notification rate of PTB, partly even with a lag effect [8, 9]. It was possible that atmospheric pollutants may affect the susceptibility of TB in individual level by the possible mechanism of inducing damage in the tracheobronchial mucosa, and triggering systemic immune responses through inhibition of the synthesis and secretion of inflammatory mediators [8]. Besides, meteorological factor such as humidity can influence the transmission of MTB in the environment, thereby altering the risk of infection in the population [6]. However, the spillover effect caused by environmental factors is denoted that these factors not only affect the local epidemic but also impact surrounding regions, which was explored for TB onset in limited literature. In addition, the pattern of communicable diseases generally showed a diversity in spatial distribution. Thus, spatiotemporal analysis has been widely used in epidemiological research to identify temporal and spatial clusters of infectious diseases. However, among the elderly, the spillover effect of environmental factors and the spatial characteristics of PTB onset remain unclear [10, 11].

Our study aimed to analyze and determine the spatial and temporal distribution characteristics and risk clustering of the elderly PTB in Zhejiang Province, eastern China, as well as to identify environmental factors that have direct and spillover effects on notification rate of PTB. These findings may provide important empirical evidence for health policy formulation and public health resource allocation.

## Methods

### Study area

**Fig. 1:**
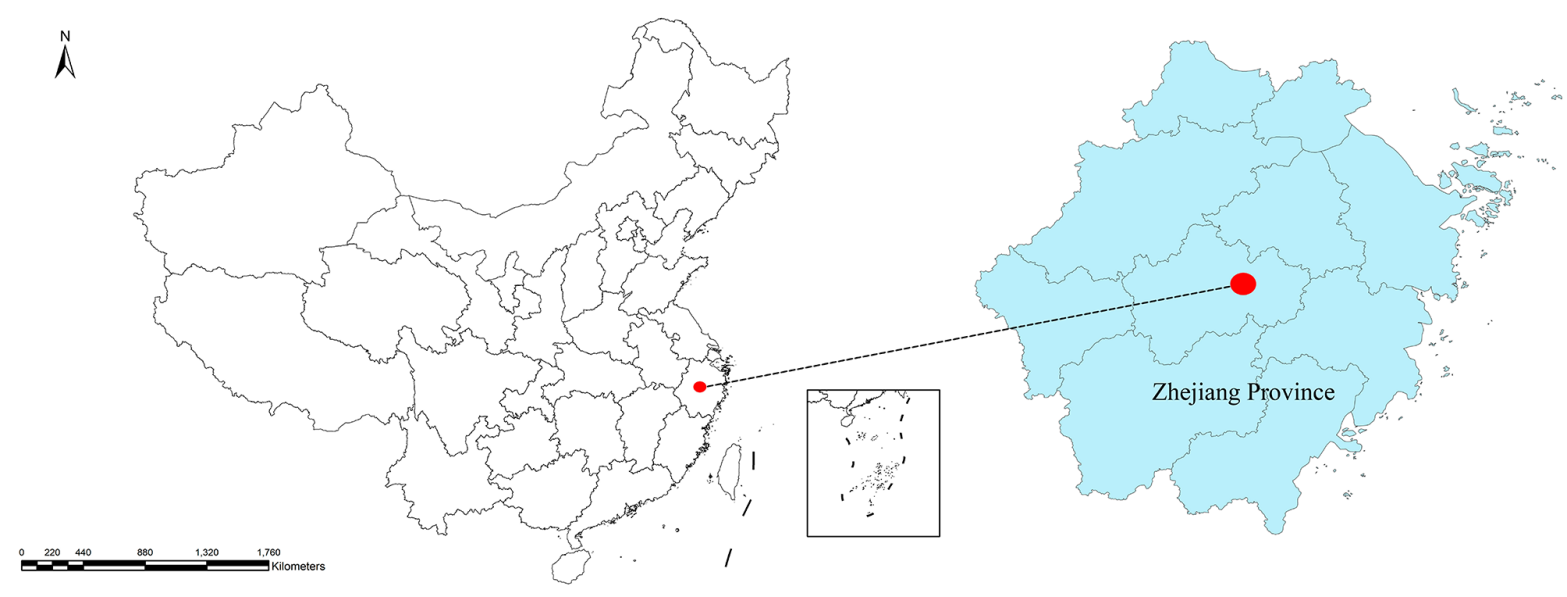
Location of the Zhejiang Province in China

Zhejiang Province is located in the eastern coastal region of China and has a total area of 100,000 km^2^ and 11 cities like Hangzhou, Ningbo, Wenzhou, Jiaxing, Huzhou, Shaoxing, Jinhua, Quzhou, Zhoushan, Taizhou, and Lishui [12]. Data from the Zhejiang Provincial Bureau of Statistics 2021 show that the resident population is 65.4 million, of which 14.2% (9.26 million) are ≥ 65 years old. The location of Zhejiang Province was mapped in Fig. 1.

### Data sources and definitions

Surveillance data of PTB cases notified from 2010 to 2021 in Zhejiang Province were collected from the Tuberculosis Information Management System (TBIMS). All included PTB cases were diagnosed and classified based on the National Diagnostic Criteria for Pulmonary Tuberculosis (WS 288–2008 and WS 288–2017) and Classification of Tuberculosis (WS 196–2017) issued by the Ministry of Health of the People’s Republic of China. PTB cases were extracted only for individuals aged ≥ 65 years with variable information of demographic and medical details; extrapulmonary TB and non-tuberculous mycobacterial cases were excluded.

Daily weather data were gathered from the China Meteorological Data Sharing Center between 2010 and 2020 (http://data.cma.cn/site). The meteorological factors collected included annual mean temperature (°C), annual mean precipitation (mm), and annual mean relative humidity (%). Air pollution data included annual mean particulate matter with diameter of less than 2.5 μm (PM_2.5_, µg/m^3^) and sulfur dioxide (SO_2_, µg/m^3^) by each county. PM_2.5_ and SO_2_ commonly co-exist in the atmospheric environment. High-resolution (1 km) annual PM_2.5_ data were from the Atmospheric Composition Analysis Group at Washington University in St. Louis (https://sites.wustl.edu/acag/datasets/surface-pm2-5/). The SO_2_ data were obtained from the GIOVANNI web-based application (https://giovanni.gsfc.nasa.gov), with a spatial resolution of 0.5° × 0.625°. We used the percentage of greenspace and the SHAPE_AM to represent the scale and form of greenspace. We obtained the 30 m annual land cover datasets in China from 1990 to 2021, which is the first Landsat-derived annual China land cover dataset (CLCD) on the Google Earth Engine (GEE) platform(10.5281/zenodo.5816591). The Fragstats version 4.2 programs were used to measure the percentage of greenspace and the SHAPE_AM in each county. Socio-economic factors were acquired, including Gross Domestic Product (GDP) per county and the density of population. The 2010–2020 GDP per county was obtained from the Zhejiang Statistical Yearbook. The population density was collected from the Worldpop platform (https://www.worldpop.org/) with a spatial resolution of 1 km × 1 km.

### Statistical analysis

#### Spatial autocorrelation analysis

R software (version 4.4.2) was used to visualize the general epidemic characteristics, GeoDa software (version 1.18.0) was used for spatial autocorrelation analysis. The spatial autocorrelation analysis of PTB data aimed to determine whether objects with similar attributes are gathered or scattered in the study space and identify risk regions, including global and local spatial autocorrelation [13]. Moran’s I index, calculated by constructing the queen adjacency matrix, is the classic measurement indicator of spatial autocorrelation:

$$ I=\frac{n}{{\sum }_{i=1}^{n}{\sum }_{j=1}^{n}{w}_{i, j}}\times \frac{{\sum }_{i=1}^{n}{\sum }_{j=1}^{n}{w}_{i,j}({X}_{i}-\stackrel{-}{X})({X}_{j}-\stackrel{-}{X})}{{\sum }_{i=1}^{n}{({X}_{i}-\stackrel{-}{X})}^{2}}$$

$$ {X}_{i}$$ and $$ {X}_{j}$$ represent the autocorrelation coefficient from counties $$ i$$ and $$ j$$, and $$ n$$ represented the number of counties in Zhejiang Province. The spatial weight matrix $$ {w}_{i,j}$$ = 1 represents that county $$ i$$ is adjacent to county $$ j$$, and $$ {w}_{i,j}$$ = 0 represents non-contiguous [14]. The Moran’s I Index ranged from − 1 to 1. The closer Moran’s I is to 1, the more likely it is to exhibit a strong positive spatial autocorrelation. Conversely, the closer Moran’s I is to -1, the more likely there is to be a strong negative spatial autocorrelation. If Moran’s I is 0, there is no spatial heterogeneity.

Local spatial autocorrelation was further performed when the global Moran’s I was significant (*P* < 0.05), and it used local indications of spatial autocorrelation (LISA) to reflect spatial clusters [15]. In the LISA map, “High-Low” indicated that high incidence was surrounded by low incidence, “High-High” indicates hot-spots and “Low-Low” indicates cold-spots [16]. The regional units for this study were 90 counties in Zhejiang Province.

#### Spatial-temporal scan statistic

The Kulldorff spatial-temporal scan statistic was used to analyze the PTB clusters in two dimensions of time and space, available in SatScan (version 10.1.1), free software for space-time data analysis. It imposes a cylinder around each point specifically, the base area of the cylinder varies across the area being searched, while the height of the cylinder varies across each year of the study period [17]. Based on the Poisson distribution model, we used the Monte Carlo randomization method to calculate the log-likelihood ratio (*LLR*) and relative risk (*RR*) of clusters using the actual and expected values to test statistically significant clustering under a 95% confidence interval (*CI*) and determine the effective clustering level [16]. The coordinates and radii of the clusters detected from the SatScan outputs were visualized using ArcGIS (version 10.4).

#### Spatial durbin model

The Spatial Durbin Model (SDM), which is based on the Spatial Lag Model (SLM) and Spatial Error Model (SEM), has certain advantages. It can identify the spatial spillover effect of environmental variables in neighboring regions on notification rate of PTB [18]. First, the Lagrange multiplier test (LM test) was used to determine the choice of SEM. Secondly, the likelihood ratio (LR) test was used to determine whether SEM should be transformed into SDM. Finally, using the Horsman test, we chose fixed-effects SDM [19]. Therefore, after adjusting for spatial confounding factors, we used SDM to examine the association between notification rate of PTB among the elderly population and environment factors with Stata/SE 15.0 software. The SDM has the following form:

$$ {Y}_{it}=\rho {WY}_{it}+\alpha {I}_{n}+\beta {X}_{it}+\theta {WX}_{it}+u$$

Where $$ Y$$ denotes the notification rate of PTB in the elderly population, $$ X$$ represents the independent variable including environmental and socioeconomic factors, $$ t$$ represents the year, $$ i$$ represents the county unit. ***In*** is the column vector of the elements, and $$ W$$ is the spatial weight matrix. The spatial weight matrix used in this study is the queen adjacency matrix based on the geographical adjacency relationship. The error term is represented by $$ u$$ and $$ \rho $$, $$ \alpha $$, $$ \beta $$, $$ \theta $$ are the influencing coefficients, respectively.

## Results

### Description of general characteristics

**Fig. 2:**
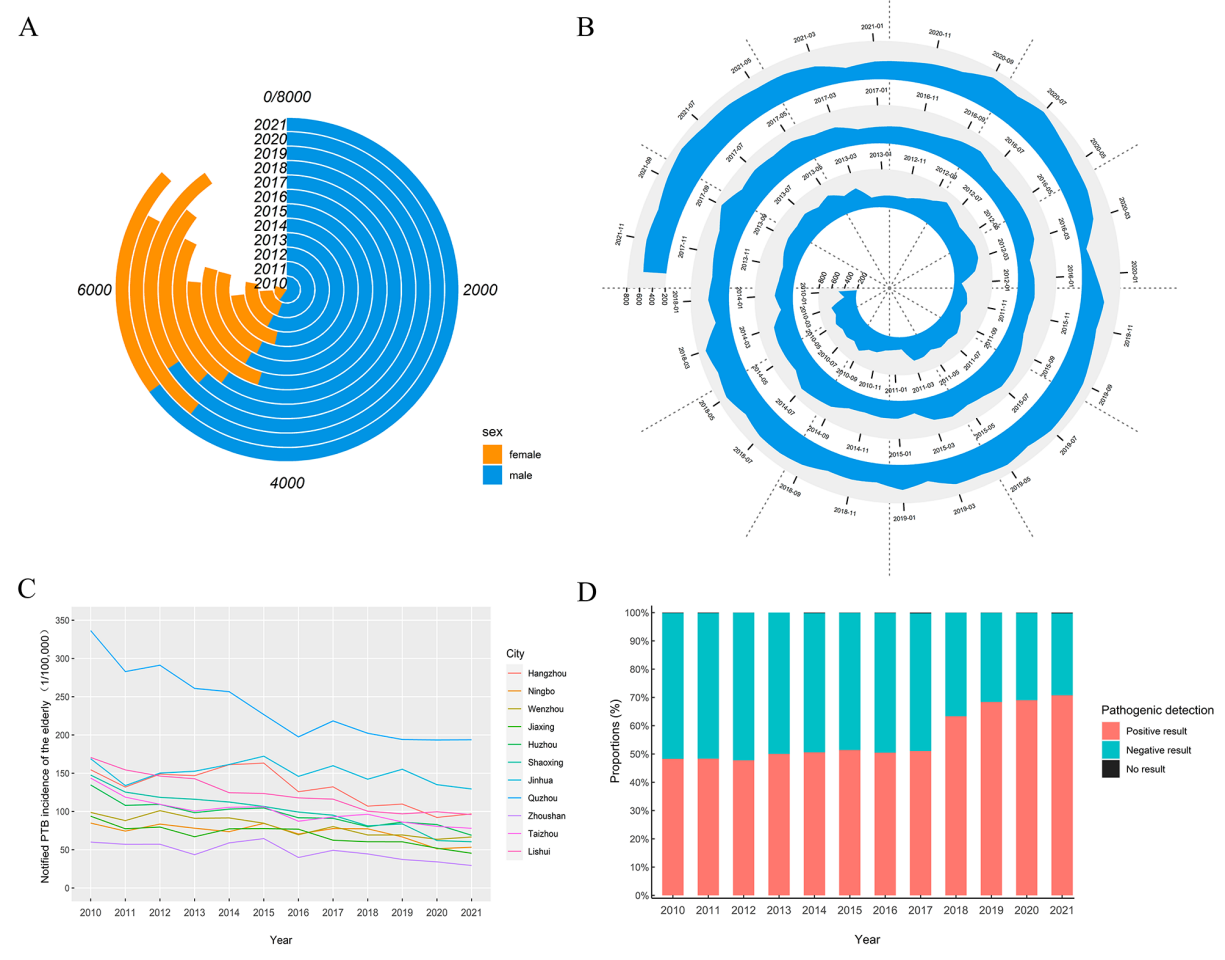
General epidemiological characteristics of PTB among the elderly in Zhejiang Province, 2010–2021. Notes: (**A**) Number of cases by sex each year; (**B**) The monthly fluctuation of PTB cases; (**C**) Changes in notification rate in 11 cities; (**D**) Proportion of pathogenic results per year

From 2010 to 2021, 77,405 PTB cases among the elderly were notified in Zhejiang Province. A total of 45,492 cases (58.8%) were laboratory-confirmed and 31,913 (41.2%) were clinically diagnosed. The notification rate of PTB has dropped from 138.67 cases per 100,000 to 81.22 per 100,000, showing an overall decreasing trend. Among the number of annual cases, the highest was in 2019 (*n* = 7218) and the lowest in 2013 (*n* = 5857), with more male than female PTB cases each year. Males accounted for 73.3% (56,726 cases) (Fig. 2A). The number of monthly PTB cases showed a periodic trend, with more cases in the summer and autumn and fewer in winter, especially in February (4,940 cases, 6.4%) (Fig. 2B). The top three cities in Zhejiang Province according to notification rate of PTB were Quzhou, Jinhua, and Hangzhou, with more than 100 cases per 100,000 people in at least 10 years. The city of Quzhou had the fastest decline in cases (Fig. 2C). Since 2018, the number of pathogen-positive cases has significantly exceeded the number of pathogen-negative cases, especially in 2021, with 4924 cases (70.8%) of pathogen-positive (Fig. 2D). Regarding age distribution, patients aged 65–69 are the most (29.5%, 22,854 cases). In terms of diverse source, 78.9% (61,093 cases) came from referral and passive cases finding and 84.9% (65,718 cases) of all patients were residents of local. In terms of occupation among PTB cases, the top three notable occupations were farming (73.4%, 56,817 cases), retired workers (14.7%, 11,405 cases), and a combination of domestic, housework, and waiting for work (8%, 6,191 cases).

Environmental factors between 2010 and 2020 for annual PM_2.5_ concentrations showed an overall decreasing trend. However, during this period, SO_2_ concentrations and temperature showed an overall increasing trend while precipitation showed an increasing trend until 2013 followed by a subsequent decreasing trend. Regarding socioeconomic factors, the GDP per county was increased as population density increased.

### Spatial autocorrelation analysis

**Fig. 3:**
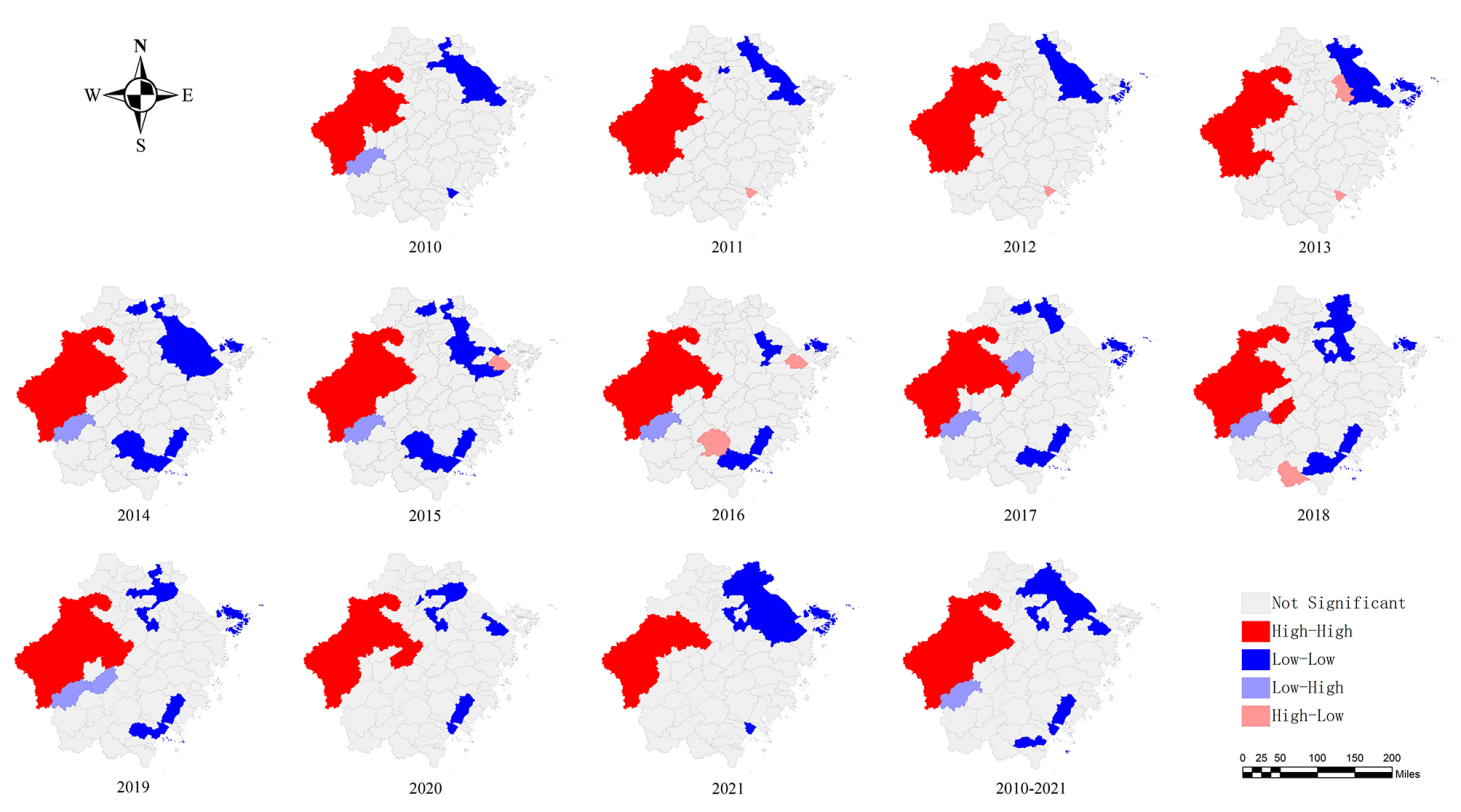
Local spatial autocorrelation of notification rate of PTB among the elderly population in Zhejiang, 2010–2021

A spatial autocorrelation analysis was conducted on the notification rate of PTB among the elderly in Zhejiang Province from 2010 to 2021. The results showed that the global Moran’s I statistic ranged from 0.522 to 0.695, with a higher statistical significance each year (*P* < 0.01) (Table 1). This indicates that the elderly PTB in Zhejiang Province was not randomized and showed significant spatial heterogeneity and positive spatial correlation at the county level. The map of the LISA cluster showed that the hotspot regions were mainly concentrated in the western part of Zhejiang Province, including parts of Quzhou, Jinhua, Hangzhou and Lishui. Although hotspot regions change dynamically annually, several counties (e.g., Jiande, Tonglu, Chun’an, Kecheng, Qujiang, Jiangshan, Changshan, and Kaihua) remained hotspots throughout the study period. In addition, during the study period, the coldspot regions changed dynamically, and their coverage was gradually expanded (Fig. 3).

**Table 1:** Spatial autocorrelation analysis of PTB notification rate among the elderly population in Zhejiang, 2010–2021

Year	Moran’s I index	Z-score	*P*-value
2010	0.525	8.246	0.001
2011	0.547	8.454	0.001
2012	0.527	8.277	0.001
2013	0.522	7.980	0.001
2014	0.591	8.698	0.001
2015	0.573	8.369	0.001
2016	0.572	7.278	0.001
2017	0.597	8.663	0.001
2018	0.624	9.138	0.001
2019	0.695	9.671	0.001
2020	0.578	8.435	0.001
2021	0.620	9.267	0.001
2010–2021	0.675	9.717	0.001

### Spatiotemporal scan statistic

**Fig. 4:**
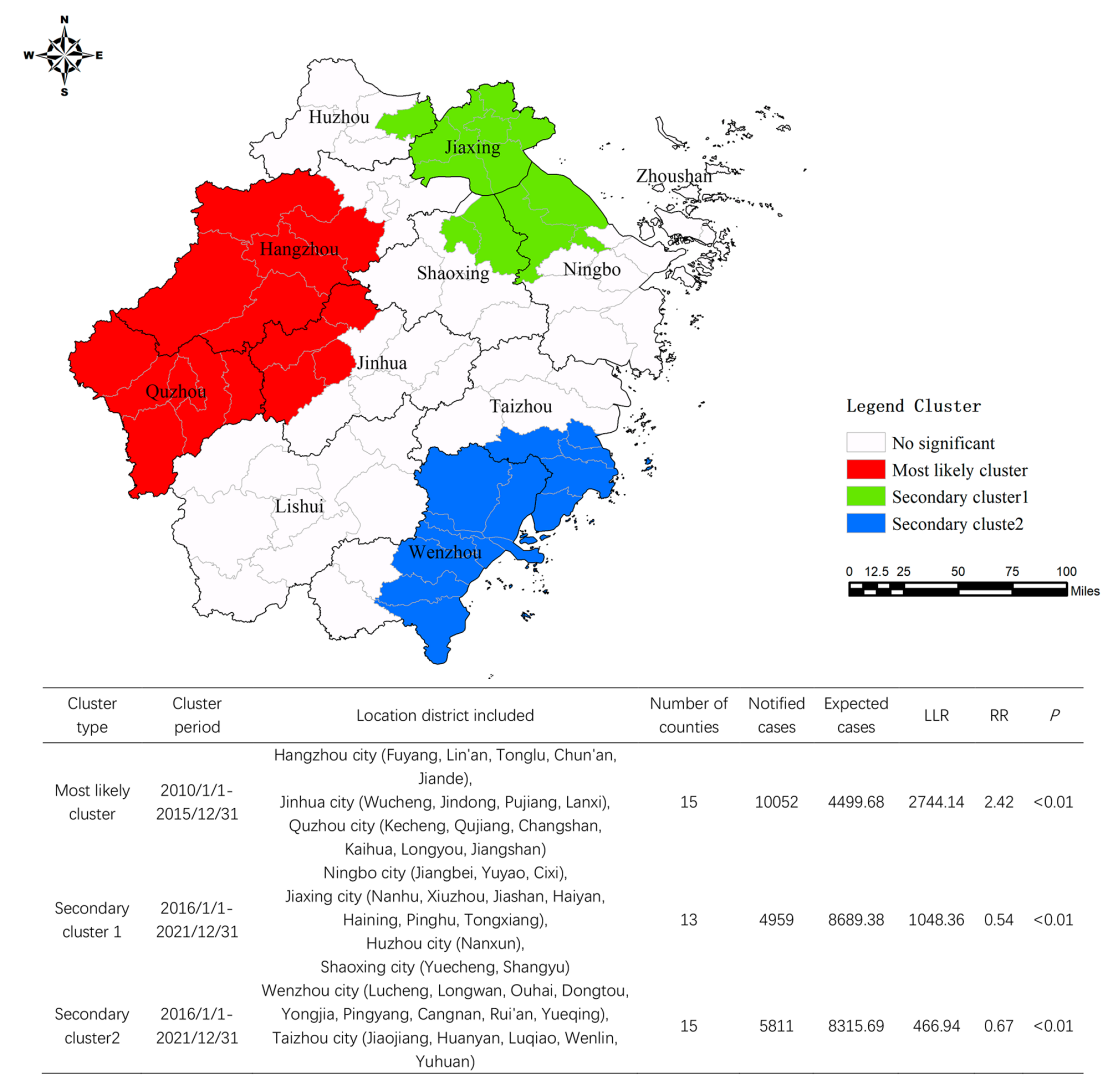
Spatial–temporal clustering results of PTB among elderly people in Zhejiang Province, 2010–2021

The spatial-temporal cluster of notified PTB cases in Zhejiang Province was determined using a spatiotemporal scanning method. During the 12-year study period, one most likely cluster and two secondary clusters were identified. The most likely cluster was high-risk regions at the specific period, in which the number of PTB cases observed was significantly higher than expected (*LLR* = 2744.1, *RR* = 2.4, *P* < 0.001). The highest risk regions located in the western part of the province, including five counties of Hangzhou (Lin’an, Fuyang, Jiande, Chun’an and Tonglu), six counties of Quzhou (Qujiang, Kecheng, Jiangshan, Changshan, Kaihua and Longyou), and four counties of Jinhua City (Jindong, Wucheng, Lanxi and Pujiang). The other two secondary clusters were protective clusters (*RR* < 1, *P* < 0.001) (Fig. 4).

### Spatial durbin model analysis

All SDM models presented in this study used time-fixed effects. The results showed that annual mean precipitation had significantly positive direct effects and spatial spillover effects on the notification rate of PTB among the elderly. Each unit increase in precipitation rised the notification rate in the local and neighboring areas by 0.167% and 0.287%, respectively. The direct effects and spatial spillover effects of annual mean temperature on notification rate were negative at the 5% level. The direct effect of annual mean relative humidity was significantly negative on the occurrence of PTB while the direct effect of PM_2.5_ was positive. A one-unit increase in PM_2.5_ led to a 0.396% increase in the notification rate. We found no direct effect of SO_2_ on the notification rate of PTB in this populationbut existing a significantly negative spatial spillover effect. Moreover, the percentage of greenspace did not affect the notification rate among the elderly; however, the SHAPE_AM was negatively associated with the rate in neighboring counties (Table 2).

**Table 2:** Spatial Durbin Model results of environmental factors and the development of PTB

Dependent Variables	SDM				
Notification rate of PTB	(1) Main	(2) Wx	(3) Direct effect	(4) Indirect effect	(5) Total effect
Annual mean precipitation	0.149*	0.177*	0.167**	0.287***	0.454***
	(0.084)	(0.094)	(0.082)	(0.102)	(0.059)
Annual mean temperature	-0.327***	-0.160	-0.349***	-0.329***	-0.678***
	(0.092)	(0.109)	(0.084)	(0.109)	(0.067)
Annual mean relative humidity	-0.168**	-0.037	-0.167**	-0.114	-0.281***
	(0.082)	(0.103)	(0.075)	(0.110)	(0.085)
PM_2.5_	0.389***	0.003	0.396***	0.149	0.545***
	(0.103)	(0.128)	(0.096)	(0.139)	(0.109)
SO_2_	0.068	-0.382***	0.0445	-0.470***	-0.426***
	(0.098)	(0.119)	(0.086)	(0.122)	(0.085)
PLAND	0.065	0.035	0.072	0.070	0.142
	(0.078)	(0.177)	(0.076)	(0.222)	(0.248)
SHAPE_AM	-0.035	-0.317***	-0.058	-0.427***	-0.485***
	(0.047)	(0.089)	(0.045)	(0.106)	(0.095)
GDP per county	-0.159***	-0.101	-0.171***	-0.185*	-0.356***
	(0.051)	(0.086)	(0.044)	(0.100)	(0.097)
the density of population	-0.008	0.003	-0.004	-0.008	-0.012
	(0.053)	(0.151)	(0.052)	(0.192)	(0.207)
ρ	0.289***				
	(0.035)				
σ	0.444***				
	(0.020)				
R^2^	0.311				
SEM-LM	284.742***				
SEM-Robust LM	1.706				
LR test (SDM & SEM)	87.220***				
SLM-LM	294.023***				
SLM-Robust LM	10.987***				
Observations	979				
Hausman test	92.410***				

Note: ***, **, and * indicate significance at 1%, 5%, and 10% levels, respectively. The standard errors are indicated in parentheses

### Sensitive analysis

To assess the robustness of the SDM model, we performed additional analyses using the Wald test, and the results showed that the model fitted well and had good robustness (Table 3).

**Table 3:** Robustness tests of the Spatial Durbin Model

Influence coefficient	Coefficient of spatial spillover
chi2(9) = 29.83	chi2(9) = 32.86
Prob > chi2 = 0.0005***	Prob > chi2 = 0.0002***

Note: ***, **, and * indicate significance at 1%, 5%, and 10% levels, respectively

## Discussion

With the challenges of an aging population, the burden of PTB in China has persisted as a public health concern [3]. This study described the general characteristics and spatiotemporal distribution, and explored the influence of environmental factors on the occurrence of PTB among the elderly. These findings may inform future targeted interventions for PTB prevention and control for this high-risk population as well as promoting the allocation of health resources.

In this study, the notification rate of PTB among the elderly population was decreased, which was consistent with the general population in the province [15]. Reasons for this decrease are likely multifactorial but may be mostly due to the implementation of DOTS and the National TB Control Program, which include strengthening government dominance, refining public health systems, improving case notification, and increasing the financial budget for PTB in Zhejiang province, China. In addition, this trend may be partly attributed to improvements at the provincial-level in pathogenic diagnosis using new technology, such as use of GeneXpert MTB/RIF, since 2017. This method allows detection of the MTB complex in less than two hours, promoting early identification and treatment of PTB cases and reducing the transmission of PTB among elderly people [20]. This also explains the successive increase in the proportion of pathogen positivity since 2017 [21]. Furthermore, the coronavirus disease pandemic led to an excessive reduction in PTB detection and case notification at early stage, which may have also caused an underestimation of the notification rate in recent years [22].

Notified PTB cases among the elderly fluctuated, with the highest number in summer and autumn and lowest in February. In winter, factors such as vitamin D deficiency due to reduced sunlight, and indoor air pollution and crowd gathering due to coldness and Chinese New Year may increase the risk of MTB infection and transmission, respectively. Thus, given the potential incubation time between infection and PTB onset and the potential delayed time, the high infection rate in the former may cause a high activated risk among the elderly in the summer and autumn [23]. Therefore, elderly people should be aware of the risk of PTB infection in winter, and public health sectors should strengthen health education to reduce clustering, strengthen nutrition, and shorten the potential delay from symptom occurrence to the behavior of health-seeking.

The spatial autocorrelation analysis indicated that the epidemics were geographically dependent. The hot- and cold-spot regions identified in the local LISA maps were similar to the most likely risk cluster and the two secondary protection clusters identified in the spatial-temporal scan statistics. Studies has shown that older adults commonly have an estimated 90% PTB due to reactivation of LTBI acquired earlier in life rather than due to recent transmission [24]. Combined with the limited economic level in the western region of Zhejiang Province, we speculated that related influencing factors such as malnutrition should be explored, and possible interventions such as vitamin A and C supplementation and standardized preventive treatment should be implemented among the latent infection population [25]. In addition, due to mass X-ray screening of people aged over 60 or 65 years, especially among the elderly with no symptoms, active cases were identified earlily and given standardized treatment regimes. This also would help decrease local PTB epidemics in the general population.

In the Spatial Durbin Model, our study provided strong evidence that meteorological factors were vital factors affecting the occurrence of PTB among the elderly. There was a significant positive association between annual mean precipitation and PTB occurrence, which was consistent with the results of Qin T et al. [26]. The spillover effect of precipitation was approximately twice as significant as its direct effect, possibly due to Zhejiang Province’s coastal location, where frequent air currents aid in the formation and dispersal of droplets and suspended particles related to TB [26]. These particles can spread in all directions with the airflow, significantly impacting neighboring areas. Additionally, TB can be transmitted through various mediums, including surface water and groundwater formed by precipitation [27]. It could cause a longer distance of dissemination in space, leading to the expansion of spatial spillover effects. Furthermore, areas with higher tuberculosis notification rate often exhibited spatial clustering, further intensifying the impact of precipitation-induced spatial spillover effects [28]. Moreover, the results indicated that the high annual mean temperature could reduce the tuberculosis occurrence in local region and had a significantly negative spillover effect. It was suggested that high temperatures may stimulate the immune system’s response, leading to increased inflammation and production of immune effector molecules, thereby enhancing the ability to clear TB. The increase in local temperature reduces outdoor gatherings and activities among the elderly [29], potentially inhibiting transmission in the external environment. Hence, the increased local temperature exerts an indirect influence on surrounding areas through the spatial spillover effects. Also, our study found different effects between air pollutants and the risk of PTB occurrence among elderly. SO_2_ has no direct effect on the notification rate of PTB. Previous study found no significant correlation between SO_2_ and the risk of TB when long-term exposure or exposure to abnormally high concentrations of pollutants was ignored [30, 31], which is consistent with our findings. However, SO_2_ has a notable negative spatial spillover effect on the health of the elderly. One potential explanation is that low-level SO_2_ exhibits antimicrobial properties by reacting with enzymes and proteins within the cell membranes of microorganisms, thereby disrupting their structure and function, leading to the inhibition of microbial growth and reproduction [32]. Therefore, considering the distance-decay-effect of SO_2_, short-term exposure to low-level SO_2_ exhibited an protective effect on the elderly in the surrounding region during the diffusion process [33]. Exposure to PM_2.5_ may increase the risk of PTB among the elderly in the local population which is consistent with previous studies [34]. Interestingly, there was no spatial spillover effect between PM_2.5_ and the tuberculosis occurrence in our study. This may be attributed to the “Low-low” distribution of air pollutants in Zhejiang province, as well as the special geographical location and meteorological conditions near the sea, which help dissipate PM_2.5_ [35]. Additionally, greenspace can contribute to the dispersal of PM_2.5_ through deposition and filtration [36], thereby reducing the negative impact of PM_2.5_ on the health of the elderly in the surrounding counties.

Despite its strengths, this study had some limitations. First, like other surveillance data, some PTB cases among the elderly may not be notified owing to a delay in seeking health care or not visiting medical institutions. Underestimation of the PTB notification rate in this specific population was unavoidable. Second, in 2019 and 2021, the administrative regions of several counties in Zhejiang Province had changed, and we integrated adjacent regions as a whole, which might ignore the spatial-temporal correlation within the integrated region. Third, the environmental factors obtained in this study were from the value of annual mean between 2010 and 2020, which may have affected the further analysis in 2021 and ignored the lag effect, leading to potential bias.

## Conclusion

Targeted interventions among the elderly in Western Zhejiang may be more efficient than broad, province-wide interventions. Decreasing environmental pollution levels, such as PM_2.5_, and enhancing the diversity of greenspace would be beneficial in controlling PTB occurrence while the low annual mean temperature and high annual mean precipitation in local and neighboring areas tend to have higher PTB onset among the elderly.

## Data Availability

All data and materials were included in this paper. The corresponding author (Bin Chen)can provide data upon reasonable request after all studies and sub-studies have been completed.

## Abbreviations

TB: Tuberculosis
PTB: Pulmonary Tuberculosis
TBIMS: Tuberculosis Information Management System
MTB: Mycobacterium Tuberculosis
DOTS: Directly observed treatment and short-course
LISA: Local indications of spatial autocorrelation
LLR: Log-likelihood ratio
RR: Relative risk
CI: Confidence interval
CLCD: China land cover dataset
GEE: Google Earth Engine
PLAND: Percentage of greenspace
SHAPE_AM: Complexity of the shape of greenspace
PM_2.5_: Particulate matter with diameter of less than 2.5 μm
SO_2_: Sulfur dioxide
CLCD: China land cover dataset
GEE: The Google Earth Engine
GDP: Gross Domestic Product
SDM: Spatial Durbin Model
SLM: Spatial Lag Model
LM test: Lagrange multiplier test
SEM: Spatial Error Model
ZJCDC: Zhejiang Provincial Center for Disease Control and Prevention
